# Systematic analysis of short internal indels and their impact on protein folding

**DOI:** 10.1186/1472-6807-10-24

**Published:** 2010-08-04

**Authors:** RyangGuk Kim, Jun-tao Guo

**Affiliations:** 1 Department of Bioinformatics and Genomics, College of Computing and Informatics, University of North Carolina at Charlotte 9201 University City Blvd, Charlotte, NC 28223 USA

## Abstract

**Background:**

Protein sequence insertions/deletions (indels) can be introduced during evolution or through alternative splicing (AS). Alternative splicing is an important biological phenomenon and is considered as the major means of expanding structural and functional diversity in eukaryotes. Knowledge of the structural changes due to indels is critical to our understanding of the evolution of protein structure and function. In addition, it can help us probe the evolution of alternative splicing and the diversity of functional isoforms. However, little is known about the effects of indels, in particular the ones involving core secondary structures, on the folding of protein structures. The long term goal of our study is to accurately predict the protein AS isoform structures. As a first step towards this goal, we performed a systematic analysis on the structural changes caused by short internal indels through mining highly homologous proteins in Protein Data Bank (PDB).

**Results:**

We compiled a non-redundant dataset of short internal indels (2-40 amino acids) from highly homologous protein pairs and analyzed the sequence and structural features of the indels. We found that about one third of indel residues are in disordered state and majority of the residues are exposed to solvent, suggesting that these indels are generally located on the surface of proteins. Though naturally occurring indels are fewer than engineered ones in the dataset, there are no statistically significant differences in terms of amino acid frequencies and secondary structure types between the "Natural" indels and "All" indels in the dataset. Structural comparisons show that all the protein pairs with short internal indels in the dataset preserve the structural folds and about 85% of protein pairs have global RMSDs (root mean square deviations) of 2Å or less, suggesting that protein structures tend to be conserved and can tolerate short insertions and deletions. A few pairs with high RMSDs are results of relative domain positions of the proteins, probably due to the intrinsically dynamic nature of the proteins.

**Conclusions:**

The analysis demonstrated that protein structures have the "plasticity" to tolerate short indels. This study can provide valuable guides in modeling protein AS isoform structures and homologous proteins with indels through placing the indels at the right locations since the accuracy of sequence alignments dictate model qualities in homology modeling.

## Background

Sequence insertions/deletions (indels) occur during evolution and alternative splicing (AS) process in eukaryotes. The generation of various protein isoforms through alternative splicing has been considered as one of the major evolutionary mechanisms for increasing the proteome size and functional diversity [1,2]. Recent high-throughput analysis based on mRNA-SEQ data from diverse human tissue and cell lines suggested that alternative splicing is almost universal (up to 94%) in human multi-exon genes [3]. While there are several types of splicing events that result in different splice isoforms when compared to the primary sequences, such as truncation, substitution, insertion and deletion, the internal insertion/deletion cases are the dominant form of alternative splicing variants and are of great interest due to its potential impact on the folding and stability of isoform structures [3,4]. In addition, genes containing "switch-like" exons are more likely to have isoforms with indels [3]. It is critical to our understanding of the function of alternatively spliced protein isoforms if we know how sequence changes, especially sequence insertions and deletions, affect the structure of the splice variants as structures hold key information for the function of proteins.

Our current knowledge about how alternative splicing affects protein structures is very limited. While there are about 28,000 annotated protein isoforms from recent UniProt release 15.11 (November 24, 2009) [5] and over 60,000 protein structures deposited in Protein Data Bank (PDB) [6], fewer than 10 pairs of alternatively spliced isoforms have documented structures [7]. Prediction of isoform structures generally falls into the category of homology modeling. However, homology modeling of proteins with indels is not a trivial task. The key to the success in homology modeling with indels is alignment accuracy, especially the positioning of the insertion or deletion sequences. For example, several groups at CASP8 (the 8th Community Wide Experiment on the Critical Assessment of Techniques for Protein Structure Prediction) used the same protein 2G39 as the template to model target protein T0438, but only three of nine models placed the insertion sequence (12 amino acids) in the right place [8]. Another infamous/famous example in indel positioning is the modeling of the long AS isoform of Piccolo C_2_A domain that has a nine-residue insertion in a loop. Instead of folding as part of the loop, the nine-residue insert displaces a β-strand that is pushed into the calcium-binding region through local rearrangement, leading to a dramatic change in calcium binding affinity [9].

While it is generally believed that insertions and deletions are well tolerated in loops [10,11], insertions and deletions within secondary structures (α-helices and β-sheets) may have a dramatic effect on the overall structure and are considered deleterious and unfavorable during evolution [4,12]. Tress *et al*. argued that AS isoform is probably an unlikely route to increase functional diversity due to probably large structural impact induced by indels [4]. Yet in a number of studies with genetically engineered insertions and deletions on T4 lysozyme, Matthews' group showed that the protein has structural plasticity to tolerate indels within secondary structures [13-15]. Three recent large scale analyses also offered a similar view that protein structures have some degree of "plasticity" to tolerate insertions and deletions through maintaining the same structural fold [16-18].

The major goal of this paper is to investigate the impact of short internal indels (less than 40 amino acids) on protein structures, especially for indels within secondary structures. Large indels may fold as an individual domain or the protein pairs may adopt different folds due to the large differences between two sequences [8]. Terminal indels are not considered in this study as terminal fragments are relatively flexible and terminal deletion/truncation have become a standard protocol in recombinant protein expression and protein crystallization [19-21]. In addition, the widely-used cloning vectors with His-tags introduce sequence artifacts that are included in the PDB SEQRES records and determining the exact tag sequences is problematic [22,23]. Though there are several similar surveys about indel statistics since 1992 [10,24-27], our approach is different as our goal is to study the impact of indels on protein structure and to provide guidance for isoform structure prediction. Therefore it is critical that the locations of the indels are unique and unambiguous. Otherwise the structural changes would be less well defined. For example, it is not uncommon that two proteins with high global sequence identity have a low local sequence similarity [28]. To address this issue, we take local sequence similarity into account and only consider protein pairs with both high global and local (sequences flanking the indels) sequence similarity (>75%) to ensure the uniqueness of indel sequences and positions. In addition, we include the "disordered conformation" in our structural analysis. It has been demonstrated that intrinsically disordered or unstructured regions are responsible for many important cellular functions and a link between alternative splicing and protein intrinsic disorder has been recently reported [17,29,30].

In this paper, we report a systematic analysis of a large non-redundant indel dataset with highly homologous protein pairs. Previously we found that the immunoglobulin (Ig) family, rich in certain amino acids including tyrosine, glycine, and serine in the third complementarity-determining region of the Ig heavy chain (CDR-H3), was overrepresented in an indel dataset [28,31,32]. Therefore those Ig-related indel sequences are not considered in our current analysis. Our results show that internal indels tend to have less regular secondary structures (α-helices and β-strands), but are rich in "disordered conformation", which is in line with the work by Romero *et al *[17]. Our data also show that proteins with short indels, including the ones within regular secondary structures, generally preserve the structural fold with some local structure rearrangement and refolding presumably for structural stability and functionality. The source of the indel, either naturally occurring or experimentally engineered, are described and the statistical significance of the features from natural indels is discussed. A webserver SCINDEL http://bioinfozen.uncc.edu/scindel was developed for convenient visualization of indel induced structural changes.

## Methods

### Generation of a non-redundant dataset of short internal indels

**Figure 1:**
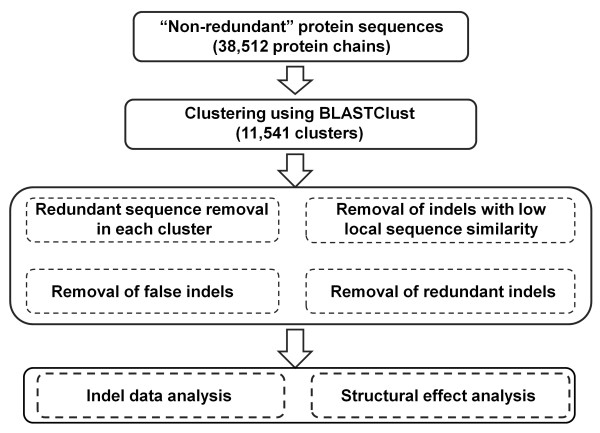
**Flowchart for indel identification and structural analysis**.

The flowchart for identification of indels from highly homologous protein pairs is shown in Figure 1. We started with a list of 38,512 protein chains from PISCES "pdbaanr" dataset (June, 2009) that contains representative protein chains selected based on the resolution and R-values [33]. The initial list of protein chains were further processed to remove NMR structures and X-ray structures with low resolution (>3.5 Å), which resulted in 31,206 protein chains. BLASTClust was used to group the protein chains into 11,541 clusters with cutoffs set at 50% and 40% for sequence similarity and alignment coverage respectively [34]. The sequence of each protein chain is derived from the SEQRES record in PDB. The purpose of this clustering step is to reduce the number of pairwise comparisons needed to find the indel sequences. A non-redundant indel dataset was generated after four filtering steps and was then subjected to statistical analysis, such as amino acids composition, secondary structure types, relative solvent accessibility, and local/global structural changes induced by the indels. Here we briefly describe the details of these filtering steps for generation of a non-redundant indel sequence dataset.

**Figure 2:**
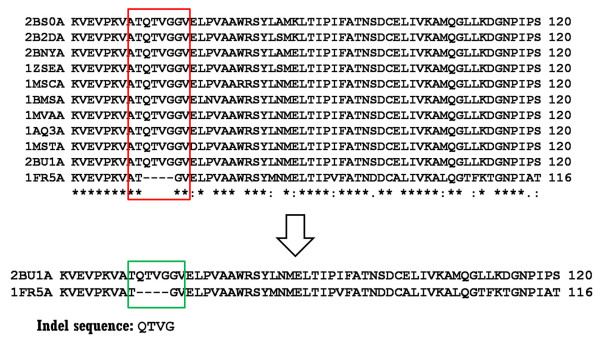
**An example for removing redundant protein chains in each cluster**.

It is possible that two or more sequences in each cluster are redundant as shown in Figure 2 in which there is only one unique indel sequence instead of 10 redundant ones from 10 pairwise alignments. Therefore the first step is to remove such redundant protein chains in each cluster that has at least two members using a similar approach as described by Pascarella and Argos [10]. Briefly, if two sequences are highly similar with no internal gaps in the alignment, the one with lower resolution is removed from the cluster. The Needle program in the EMBOSS package, an implementation of the Needleman-Wunsch global alignment algorithm, was used for sequence alignment with default parameters [35,36].

The second step of the procedure is to ensure the uniqueness of indel sequences and locations by checking the local sequence similarity. Two proteins with high global sequence identity may have regions that show low local sequence similarity [28]. If an indel happens to be in the low sequence similarity area, the placement of this indel may change dramatically with minor changes of alignment parameters, resulting in different indel sequences and locations. In our approach, similarities of the sequences flanking the indels (20 amino acids on each side) were calculated. We only consider indel sequences from protein pairs with both high global sequence similarity and highly similar flanking regions (above a cutoff value of 75%).

**Figure 3:**
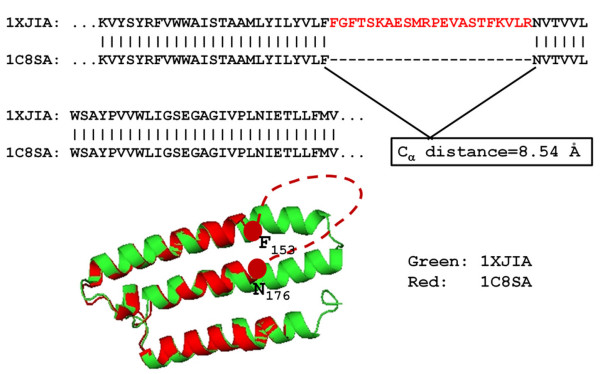
**An example of a false indel sequence derived from 1XJIA-1C8SA**. Dashed red line represents the disordered fragment. The Cα distance between the two residues (F and N) that flank the disordered fragment in 1C8SA is 8.54 Å.

Due to the discrepancies of deposition of SEQRES in PDB by experimental structural biologists, some indels derived from the SEQRES sequence comparisons are not true indels, especially in the cases of disordered fragments. For example, the sequence alignment between proteins 1XJIA and 1C8SA of the same protein, bacteriorhodopsin, shows an internal gap based on the SEQRES sequences (Figure 3). However, the fragment 154-175 in 1C8SA is disordered in the X-ray structure and the sequence of this fragment was not reported in the SEQRES. The same fragment adopts an α-helical structure in 1XJIA and appears in the SEQRES record. These types of fragments that adopt ordered conformation in one structure and are disordered in another structure have been dubbed as "dual personality" fragments [37]. If the disordered fragments were not reported in SEQRES section, such "dual personality" fragment would introduce false indels. A simple way to identify the false indels is to check the C_α _distance between the two indel flanking residues in the short form. If the two residues are connected, the C_α _distance should be around 3.85Å (data not shown). An indel is flagged as false if the C_α _distance is more than a cutoff value (4.5Å in this study). In the above example, the C_α _distance between F_153 _and N_176 _in 1C8SA is 8.54Å. Therefore this indel of 22 residues was flagged as a false one and was removed from the dataset (Figure 3).

Lastly, indel sequences were further processed to generate a non-redundant dataset of indels ranging from 2 to 40 amino acids. Two indel sequences are considered redundant if two protein pairs are from the same family and have the same indel sequences with very similar secondary structures at approximately the same residue positions.

To check if an internal indel is a result from engineered mutants or from natural variants, we combined the information from the SEQADV record of PDB files with manual inspection of related publications. The PDB SEQADV record describes conflicts between residue sequences in the ATOM/HETATM records and those in sequence databases [6]. Since there are several possible reasons for these conflicts, including engineered mutants, natural variants, disordered fragments, or cloning artifact, careful manual inspection is needed.

### Secondary structure types and relative solvent accessibility of indel residues

Each indel residue was assigned to one of four secondary structure states, helix, strand, coil and disordered. DSSP program was used to assign the first three secondary structure states: helix, strand and coil [38]. Following the widely used convention, H (α-helix), G (3_10_-helix) and I (π-helix) from DSSP are classified as helix type while E (extended strand) and B (residue in isolated β-bridge) states are classified as strand type. All the other states from DSSP are considered as coil. The disordered residues are defined by comparing the "ATOM" and "SEQRES" records in PDB file. If a residue or a fragment appears in "SEQRES", but is missing from the "ATOM" record in a PDB file, this residue or fragment is considered as disordered or unstructured [39]. The relative solvent accessibility was calculated by dividing the absolute value from DSSP by the maximum accessibility of each residue [40]. We employ a three-state classification for relative solvent accessibility: buried (≤7%), intermediate (>7% and ≤37%), and exposed (>37%) [41]. The disordered/unstructured residues were considered as exposed in solvent accessibility analysis.

For comparison purpose, a non-redundant data set with 4731 protein chains, in which no pair of protein chains has more than 25% sequence identity, each structure has a resolution of better than 2.5 Å, and the size is in the 50-1000 amino acids range, was used as background analysis for amino acid composition, secondary structure types, and residue solvent accessibility.

### Protein sequence and structure comparisons

Sequence alignment is done with the Needle program with default parameters [35,36]. Two different structure alignment programs, FAST [42] and CE [43], were used for global and local structure alignment respectively. The structural difference/similarity is measured by the Cα RMSD of aligned residues between two structures. The structural changes induced by indels were evaluated by comparing the structure and sequence alignments of each pair. A webserver SCINDEL http://bioinfozen.uncc.edu/scindel was developed for convenient visualization of both the sequence and structure alignments and structural changes caused by indels.

## Results and discussion

### A non-redundant dataset of short internal indels from highly homologous protein pairs

The protein chains were first clustered into 11,541 groups using BLASTClust as described in the Methods section. The first filtering step of removing redundant protein chains in each cluster resulted in 1,932 clusters that have two or more members. A total of 1,237,062 internal indels were identified from 445,552 distinct protein pairs. A dataset of 1,114 non-redundant indels were generated after removing indels that are false, redundant, or the results of low local sequence similarity. As described earlier [28], the dataset is rich in indels (931 of 1114) derived from one specific family member, immunoglobulin variable domain (b.1.1.1) based on the latest SCOP release 1.75 [44]. These indels are generally located in the CDR-H3 loops that play crucial roles in antigen recognition and binding specificity [32,45]. The CDR-H3 loops are dominated by residues tyrosine, glycine, and serine, but have fewer lysine, glutamine, and glutamic acid [28,31,32]. Due to the over-representation of indels from the immunoglobulin family, these indels were removed and the final non-redundant indel dataset includes 183 indels. The detailed information for each indel, including length, amino acid sequence, host proteins, start and end positions of the indel, and SCOP classification is available at http://bioinfozen.uncc.edu/scindel/nonredundant_indels.html.

### Statistical analysis of the indel sequences and structures

**Figure 4:**
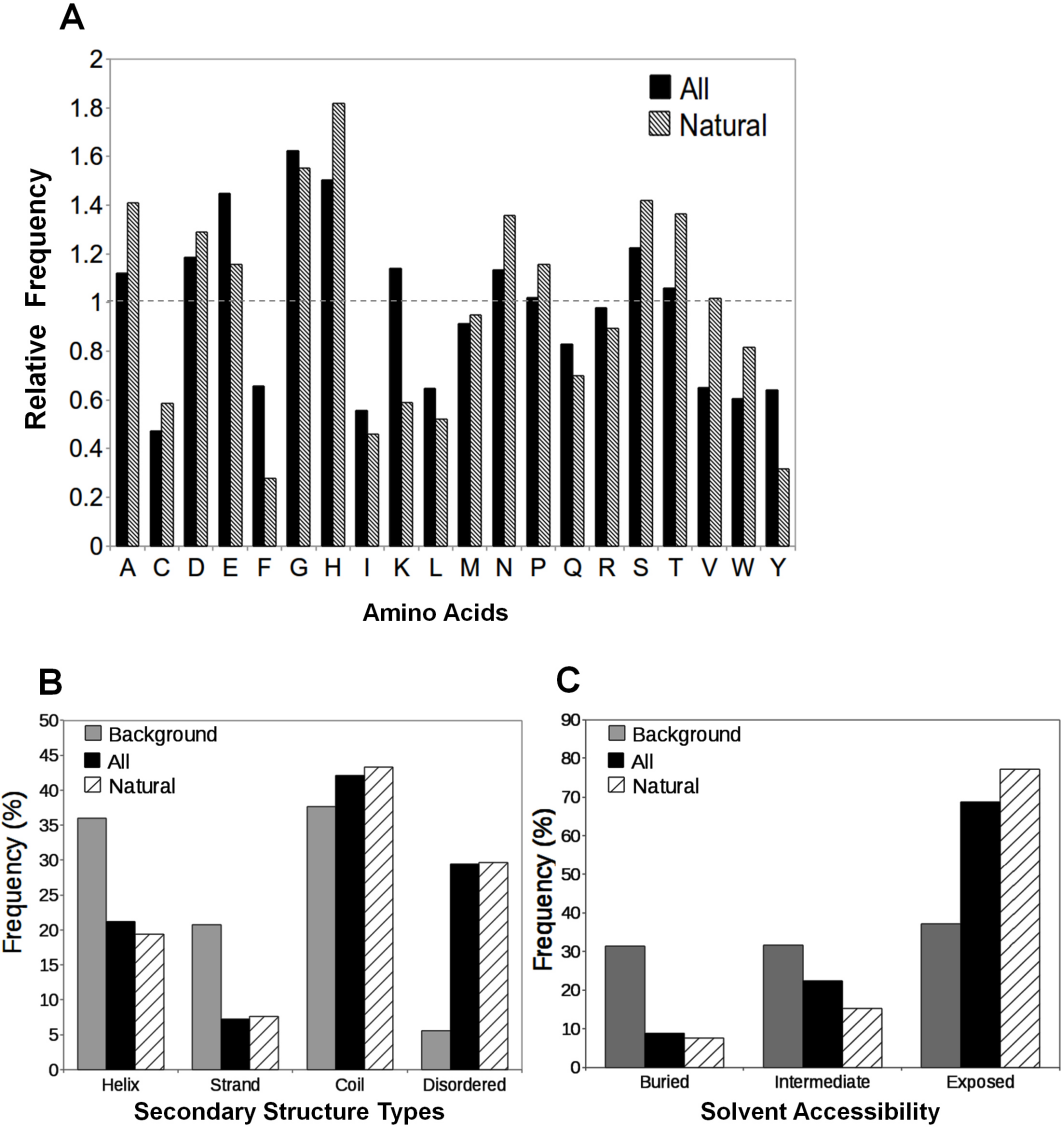
**Comparisons of amino acid compositions, secondary structure types and relative solvent accessibilities of indel residues in "all indels", "naturally occurring indels" and reference datasets**. Relative frequencies of 20 amino acids, frequencies of secondary structure types (helix, strand, coil, and disordered), and relative solvent accessibilities (buried: ≤7%, intermediate: >7% and ≤37%, exposed: >37%) are shown in A, B and C respectively. The one-letter code for amino acids is used. "Background" data for amino acid frequencies, secondary structure types and solvent accessibilities are calculated from a dataset of 4731 non-redundant protein structures (See Methods). "Natural" represents an indel dataset without engineered indels. "All" indel dataset includes both engineered and natural indel sequences.

The dataset with all short internal indels has 1301 total residues and is rich in residues glycine, histidine, glutamic acid, aspartic acid, and serine, but are depleted in residues cysteine, phenylalanine, isoleucine, leucine, tryptophan, and tyrosine when compared with the background residue frequencies ("All" in Figure 4A and Additional file 1, Figure S1). The relative frequency of each amino acid in Figure 4A is normalized with its background frequency. Residues isoleucine and leucine have high propensity to adopt α-helix or β-sheet conformations while glycine, aspartic acid, and serine prefer to be in loops. The amino acids compositions of indels suggest that indel sequences assume less regular secondary structures and prefer to be in more flexible regions, which are supported by the analysis of secondary structure types (Figure 4B). While there is a dramatic decrease in the number of residues that adopt regular secondary structures, especially the sheet conformations, the number of coil residues is only slightly more than that from the background distribution (Figure 4B). Instead, relative to the background frequencies, indel sequences have a markedly increased number of residues in disordered state (over five-fold increase) (Figure 4B). Figure 4C shows that most indel residues (~70%) are exposed to solvent. Similar observations have been reported for alternative splicing events that, by and large, prefer coil regions and exposed residues [16,17]. It should be pointed out that some disordered fragments may fold as regular secondary structures under different conditions, such as the existence of ligands or other proteins. This type of fragments that can exist in both ordered and disordered states have been termed as "dual personality" fragments [37]. Taken together, only a small percentage of the indel residues fold into regular secondary structures and are embedded inside of the proteins. χ^2 ^analysis based on the observed and expected (background frequencies as references) numbers indicates that the differences are statistically significant with very low *p*-values (Additional file 1, Table S1).

We also checked the conformation of five residues flanking the indel sequences on each side and found that more residues are in β-sheet or α-helix states when they are further way from the indel sites (Additional file 1, Figure S2).

### Source of the non-redundant indel sequences

A number of studies have been devoted to study the structural and functional consequences of insertions and deletions with artificially engineered constructs [12-15]. For example, Matthews' group has used T4 lysozyme as a model system to investigate the effect of insertions on protein structures, including an insertion in an α-helix [13,14]. The very question about the indel dataset in this study is how many of the indel sequences are from naturally occurring proteins rather than "man-made". Based on the SEQADV records of PDB files and manual inspection, we found that about 70% of the indel sequences in our dataset are the results of engineered insertion/deletion mutants. Besides the T4 lysozyme structural studies, the majority of the engineered insertion/deletion mutants were constructed to investigate the functional importance of a particular fragment. Deletion mutants have also served as one of the rational engineering approaches to better crystallization in protein structure determination [21]. Indels from protein pair 2J4OA-2POPA (19 AAs) and 2RHSB-2RHQB (4 AAs) are such examples. The indel statistics from naturally occurring indels in terms of secondary structure types and solvent accessibilities are significantly different from those of the reference dataset and are highly similar to those derived from all indels (Figures 4B and 4C). Though there are variations in amino acid frequencies between all indels and naturally occurring ones, the general trend is surprisingly similar (Figure 4A). Due to the small size of the naturally occurring indel dataset (55 indels, 263 residues), the significances of differences between the observed numbers of amino acids, secondary structure types, and relative solvent accessibility and the expected numbers (using either "Background" or "All" indels as references) were calculated with χ^2 ^test. Not surprisingly, there are significant differences between the observed numbers in the naturally occurring indel dataset and the expected numbers (based on background distributions) with *p*-values of 5.1e^-4^, 2.6e^-68^, and 9.32e^-41 ^for amino acid, secondary structure types, and relative solvent accessibility, respectively (Additional file 1, Table S2). More importantly, there are no statistically significant differences between the naturally occurring indels and the all indel dataset in terms of amino acid frequencies (*p *= 0.46) and secondary structure types (*p *= 0.91) (Additional file 1, Table S2), suggesting that the features from our all indel dataset may represent the properties of real, non-engineered indel sequences. Statistical analysis also showed that the levels of solvent accessibility are different at a significance level of 0.01 between all indels and naturally occurring indels (*p *= 0.0094), largely due to the differences in the intermediate and exposed categories (Figure 4C). Unlike secondary structure types, the classification of relative solvent accessibility into three categories is rather broad and arbitrary; so the differences may become minor if different bins or classifications are used.

### Impacts on structural changes by indels

**Figure 5:**
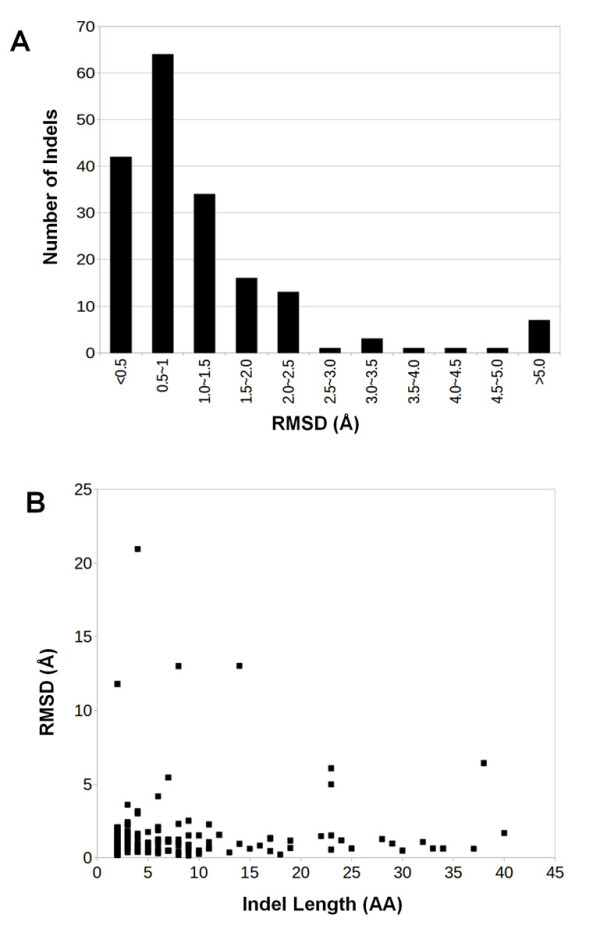
**Structural comparisons of protein pairs with indel sequences using FAST **[42]. (A) Distribution of global RMSDs; (B) relationship between global RMSDs and indel lengths.

To investigate the structural changes caused by indels, we used two structural alignment programs FAST [42] and CE [43] for global and local structural comparison respectively. Figure 5A shows the distribution of the global structure differences in terms of RMSD. Most of the structural pairs are highly similar with about 85% of the protein pairs having less than 2Å RMSDs, suggesting that these protein structures in general can tolerate and accommodate the indels [12,18]. Intuitively, the RMSDs that measure the structural differences would increase with the length of indels. However, there is no clear relationship between the RMSDs and the length of the indels (Figure 5B). Many long indels lead to minimal conformational changes, which is not surprising as majority of the indel sequences adopted either coil or disordered "conformation". This observation is consistent with the previous reports that insertions/deletions are most likely to occur in loop regions or between regular secondary structure elements and thus preserve the overall structural fold [12].

**Figure 6:**
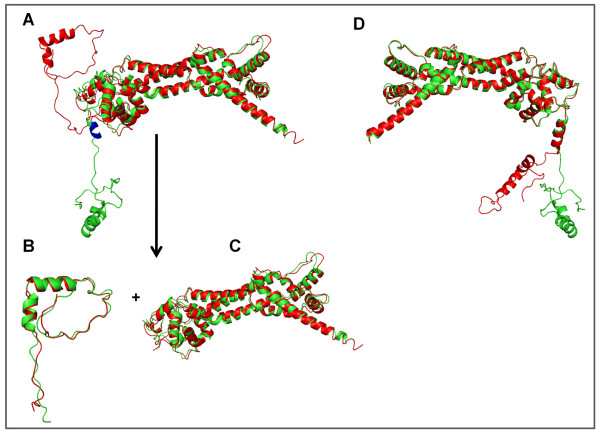
**Global and local structure alignments**. (A) Structural alignment between 1Y64B and 1UX4A; (B) and (C) N-terminal and C-terminal structural alignments, respectively; and (D) structural alignment between 1Y64B and 1UX5A. Green: 1Y64B. Red: 1UX4A in A, B, and C and 1UX5A in D.

On the other hand, several large RMSDs are associated with relatively short indel sequences (Figure 5B). We found that all the nine pairs with RMSDs of 4Å or more are the results of indels in the hinge area, causing changes in the relative orientations of the domains connected by the indels, rather than changing to different folds. For example, 1Y64B-1UX4A (with a four-residue indel sequence "REDL" folding into a helical structure) has the largest global RMSD of 20.94Å (Figure 6A). However, the N- and C-terminal domains separated by the indel sequence have almost identical structures in 1Y64B and 1UX4A, with RMSDs of 0.95Å and 1.11Å respectively (Figure 6B and 6C). The obvious question in these cases is whether the short indels induce such domain movements. It turned out that the intrinsic flexibility and dynamics of proteins may play a bigger role in the structural changes. For example, 1UX5A and 1Y64B have different domain positions though both crystal structures are from the same protein (Figure 6D).

**Figure 7:**
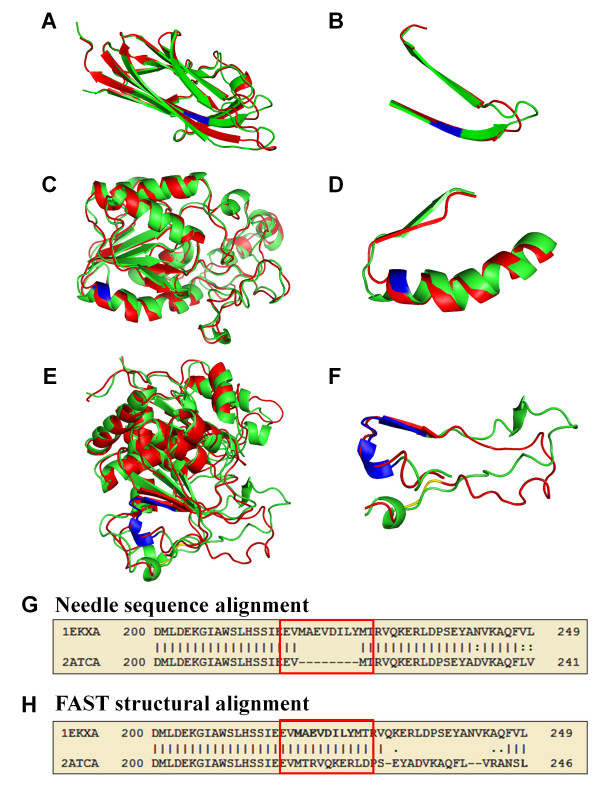
**Structural comparisons of proteins with indels adopting α-helix and/or β-strand conformations**. (A, B): 1RJ7A-1RJ8A; (C, D): 5PGMA-3PGMA; (E, F): 1EKXA-2ATCA. (A, C, E): whole structure alignments; (B, D, F): highlights of alignments in the indel region; (G): part of the sequence alignment between 1EKXA and 2ATCA involving the indel sequence MAEVDILY; (H): part of the structural alignment between 1EKXA and 2ATCA involving the indel sequence. Green: long protein; Red: short protein; Blue: indel sequence.

While it is generally accepted that insertion/deletion in loops introduces minimal structural changes, the effects of indels on regular secondary structures, especially β-strands, are greatly debated [18,46,47]. The deletion of β-strands in a β-sheet also presents a challenging problem for comparative modeling approaches. In our indel dataset, there are fifteen cases in which indels fold into α-helices or β-strands and are less exposed. In almost all cases, the core secondary structures tend to be conserved even though the shorter form lacks part of the sequence that folds as a strand or a helix in the longer form (Figure 7A-H). In these protein pairs, part of a strand (1RJ8A-1RJ7A, Figure 7A&7B), a helix (5PGMA-3PGMA, Figure 7C&7D), or a combination of strand and helix conformations (1EKXA-2ATCA, Figure 7E-H) at the sites of indels are conserved with small changes in the neighboring loop areas through local structure rearrangement and refolding. For example, the indel sequence with eight residues (MAEVDILY) from 1EKXA folds as a helix-loop-strand. In the short form of 2ATCA, the eight residues after the indel (MTRVQKER) assume the same structural conformation of the indel sequence while the downstream loop becomes shorter and deviates from the loop conformation in the long form (Figure 7E-H). The slight conformational change in the loop is not surprising as the loop is on the surface area and is flexible. Nevertheless, it shows the inherent capability of proteins to tolerate short structural deletions and insertions [18,47].

We shall point out that even though all the short indels (engineered or natural) in our dataset do not show big impact on protein structures, it does not necessary mean that short internal indels have no deleterious effect. First of all, all the proteins with natural indels in our dataset are probably the ones that survived from evolution events while the others with dramatic structural effect might have disappeared during evolution. Secondly, even in the cases where indels do not induce structural change, a disastrous loss of function may occur. Nevertheless, these data (natural and engineered indels) strongly suggest the inherent structural plasticity of protein structures [16-18].

**Figure 8:**
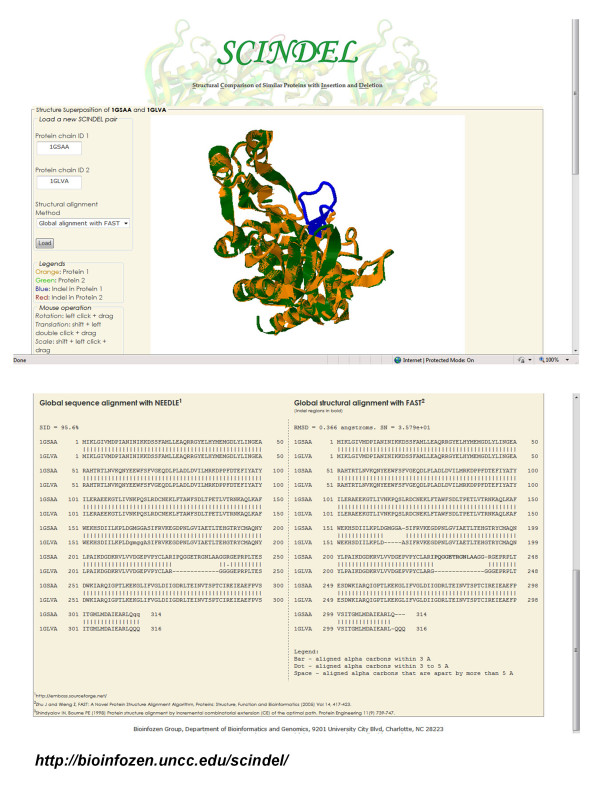
**A snapshot of SCINDEL webserver**. Sequence and structure alignments between 1GSAA and 1GLVA with an indel sequence of 13 amino acids.

All the protein pairs with indels http://bioinfozen.uncc.edu/scindel/nonredundant_indels.html can be visualized at both sequence and structural level using our SCINDEL (Structural Comparison of Similar Proteins with Insertion and Deletion) webserver at http://bioinfozen.uncc.edu/scindel. Figure 8 shows a snapshot of the comparisons between 1GSAA and 1GLVA with an indel of 13 amino acids.

## Conclusions

We performed a systematic study to investigate the impact of short internal indels on protein structures through mining the highly homologous protein pairs in PDB. In addition to protein evolution, indels can be the results of alternative splicing. We found that short internal indels tend to occur between secondary structure elements and a significant number of indels are disordered, which is in agreement with the earlier studies that demonstrated the associations among indels, structural disorder, and functional diversity [17]. The rationale of choosing highly homologous protein pairs (with high sequence identity for both overall and indel flanking sequences) is two-fold: 1) to avoid "random" positioning of indel(s) in a protein pair due to low local sequence similarity even though overall sequence similarity is high; and 2) to provide a better approximation to the AS isoforms with internal gaps (100% identical in indel flanking regions). These steps ensure unambiguous indel sequences and their unique positions, reducing the possibility of including false indels due to sequence alignment errors.

One important observation from this study is that most of the indels in the dataset are not derived from evolution events. Indels have been engineered into proteins for various purposes, including structural and functional studies of short peptides and better protein crystallization. Our statistical analysis showed that there are significant differences between naturally occurring indels and the control dataset. On the other hand, there are no statistically significant differences between naturally occurring indels and all indels in terms of amino acid frequencies and secondary structure types. These data suggest that the indel properties derived from our all indel dataset are very useful.

The very question about modeling isoform structures or structural changes due to indels is how to improve the sequence alignment for comparative modeling since the performance of current comparative modeling techniques rely heavily on accurate alignments [8]. Very rarely, sequence alignment errors can be recovered by current comparative modeling programs. We believe this systematic analysis, along with earlier reports on the case studies with individual or a small number of indel pairs, will help us in this regard as well as in our understanding of the structural plasticity of proteins.

## List of abbreviations used

PDB: Protein Data Bank; RMSD: Root Mean Square Deviation; AS: Alternative Splicing; Indel: Insertion/deletion;

## Authors' contributions

JTG designed and supervised the study and wrote the manuscript. JTG was also involved in data analysis. RGK developed the webserver and the programs for data generation, and participated in data analysis. Both authors read and approved the final manuscript.

## Supplementary Material

Additional file 1 **Figure S1: Comparison of amino acid frequencies of indel sequences in "all indels" (All), "naturally occurring indels" (Natural) and reference (Background) datasets**. Figure S2: Frequencies of secondary structure types for residues flanking indel sequences. Table S1: Statistical significance analysis of the observed numbers in "all indels" dataset. Table S2: Statistical significance analysis of the observed numbers in naturally occurring indels. Click here for file
