# Supplementary material for: Systematic analysis of short internal indels and their impact on protein folding
Source: BMC Struct Biol. 2010 Aug 4;10:24. doi: 10.1186/1472-6807-10-24 (PMC2924343; doi:10.1186/1472-6807-10-24)
Supplement: Additional file 1 — Figure S1: Comparison of amino acid frequencies of indel sequences in "all indels" (All), "naturally occurring indels" (Natural) and reference (Background) datasets. Figure S2: Frequencies of secondary structure types for residues flanking indel sequences. Table S1: Statistical significance analysis of the observed numbers in "all indels" dataset. Table S2: Statistical significance analysis of the observed numbers in naturally occurring indels. [file 1472-6807-10-24-S1.PDF]

Additional files

Figure S1. Comparison of amino acid frequencies of indel sequences in “all indels”(All), “naturally occurring indels”(Natural) and reference (Background) datasets.

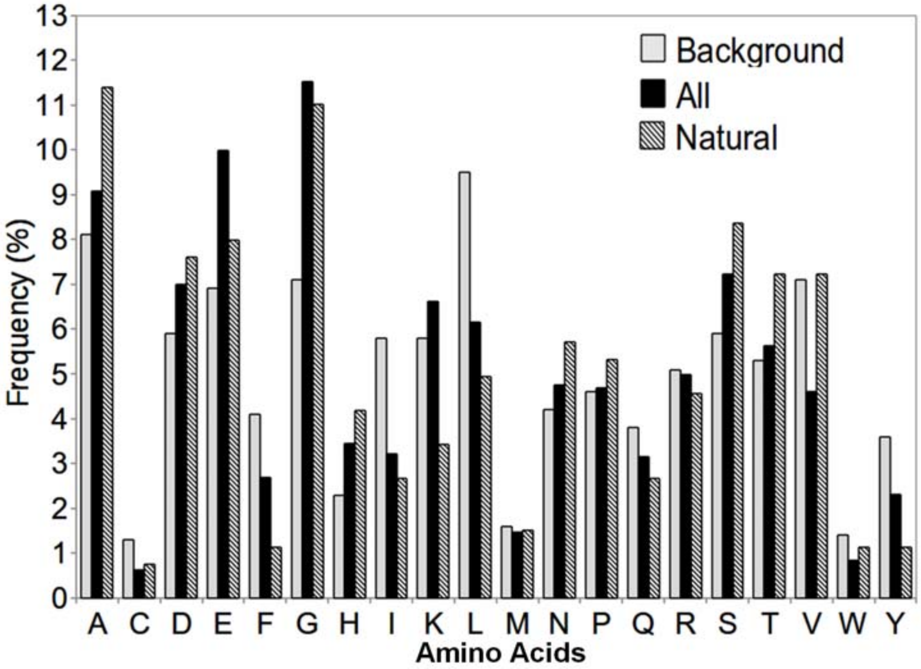

Figure S2. Frequencies of secondary structure types of residues flanking indel sequences.

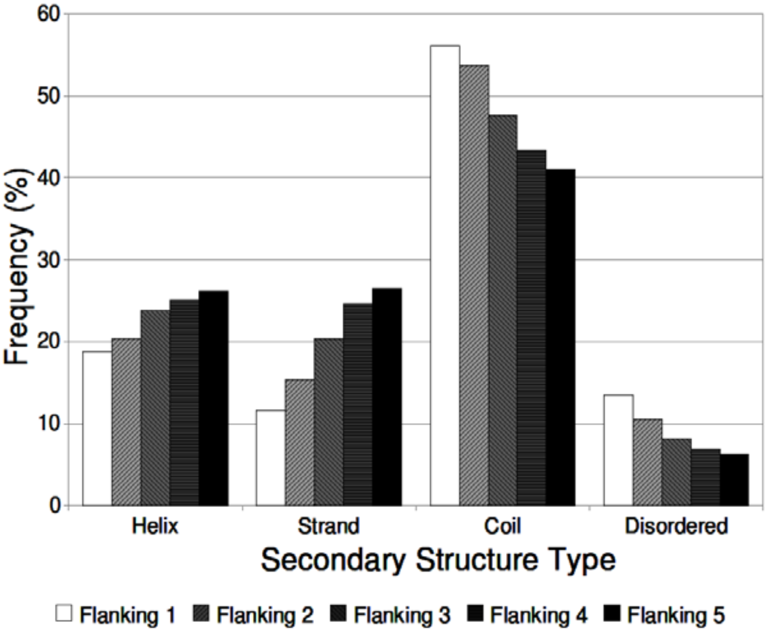

Table S1. χ^2 **analysis of amino acids frequencies, secondary structure types, and relative solvent accessibility of "All" indels**

Amino acids	A	C	D	E	F	G	H	I	K	L	M	N	P	Q	R	S	T	V	W	Y	*p-value*
**observed**	118	8	91	130	35	150	45	42	86	80	19	62	61	41	55	94	73	60	11	30	$1.36e^{-19}$
**expected**[1]	105	17	77	90	53	92	30	75	75	124	21	55	60	49	66	77	69	92	18	47	

Secondary structure types	helix	strand	coil	disordered	*p-value*
**observed**	276	94	548	383	0.0
**expected**[1]	468	270	490	73	

Relative solvent accessibility	buried	intermediate	exposed	*p-value*
**observed**	115	292	894	$1.28e^{-130}$
**expected**[1]	408	411	482	

[1]Expected numbers are calculated using the frequencies from the background dataset as described in Methods

Table S2. χ^2 analysis of amino acids frequencies, secondary structure types, and relative solvent accessibility of "Natural" indels

Amino acids	A	C	D	E	F	G	H	I	K	L	M	N	P	Q	R	S	T	V	W	Y	*p-value*
**observed**	30	2	20	21	3	29	11	7	9	13	4	15	14	7	12	22	19	19	3	3	
**expected[1]**	21	3	16	18	11	19	6	15	15	25	4	11	12	10	13	16	14	19	4	9	$5.1e^{-4}$
**expected[2]**	24	2	18	26	7	30	9	8	17	16	4	13	12	8	13	19	15	12	2	6	0.46

Secondary structure types	helix	strand	coil	disordered	*p-value*
**observed**	51	20	114	78	
**expected[1]**	95	55	99	15	$2.6e^{-68}$
**expected[2]**	56	19	111	77	0.91

Relative solvent accessibility	buried	intermediate	exposed	*p-value*
**observed**	20	40	203	
**expected[1]**	83	83	97	$9.3e^{-41}$
**expected[2]**	23	59	181	$9.4e^{-3}$

[1]Expected numbers are calculated using the frequencies in the "Background" dataset as described in Methods

[2]Expected numbers are calculated using the frequencies of "All" indels.
